# Architectural constraints are a major factor reducing path integration accuracy in the rat head direction cell system

**DOI:** 10.3389/fncom.2015.00010

**Published:** 2015-02-06

**Authors:** Hector J. I. Page, Daniel Walters, Simon M. Stringer

**Affiliations:** Departmental of Experimental Psychology, Oxford Center for Theoretical Neuroscience and Artificial Intelligence, University of OxfordOxford, UK

**Keywords:** spatial cognition, head direction cells, path integration, continuous attractor neural networks, attractor dynamics

## Abstract

Head direction cells fire to signal the direction in which an animal's head is pointing. They are able to track head direction using only internally-derived information (path integration)In this simulation study we investigate the factors that affect path integration accuracy. Specifically, two major limiting factors are identified: rise time, the time after stimulation it takes for a neuron to start firing, and the presence of symmetric non-offset within-layer recurrent collateral connectivity. On the basis of the latter, the important prediction is made that head direction cell regions directly involved in path integration will not contain this type of connectivity; giving a theoretical explanation for architectural observations. Increased neuronal rise time is found to slow path integration, and the slowing effect for a given rise time is found to be more severe in the context of short conduction delays. Further work is suggested on the basis of our findings, which represent a valuable contribution to understanding of the head direction cell system.

## 1. Introduction

Head direction (HD) cells respond to the animal's HD in the horizontal plane (Ranck, 1985; Taube et al., 1990a,b). They are influenced by both visual and self-motion (idiothetic) cues, yet are able to sustain and correctly update firing through idiothetic information alone (Goodridge and Taube, 1995; Taube, 1995; Blair and Sharp, 1996; Goodridge et al., 1998; Zugaro et al., 2002; Stackman and Zugaro, 2005), a process known as path integration (Mittelstaedt and Mittelstaedt, 1980; Etienne and Jeffery, 2004).

Most path integration models are based on a continuous attractor neural network (CANN) layer of HD cells. External input shifts a packet of activity representing current HD through the HD layer (Skaggs et al., 1995; Redish et al., 1996; Sharp, 1996; Goodridge and Touretzky, 2000; Stringer et al., 2002; Stringer and Rolls, 2006; Stratton et al., 2011). This external input originates in the output of angular head velocity (AHV) cells (Bassett and Taube, 2001; Sharp et al., 2001).

Path integration must happen at the correct speed for the system to accurately track true HD. However, the factors governing path integration speed have not been fully investigated. One theory of how time-accurate path integration is achieved (Walters et al., 2013) uses the idea of forming associations over axonal conduction delays naturally found in the brain. This strengthens connections between a starting HD and a predicted HD after head rotation at a particular speed over a known delay. During path integration, this connectivity shifts activity through the HD layer.

### 1.1. Hypothesized path integration mechanism

**Figure 1:**
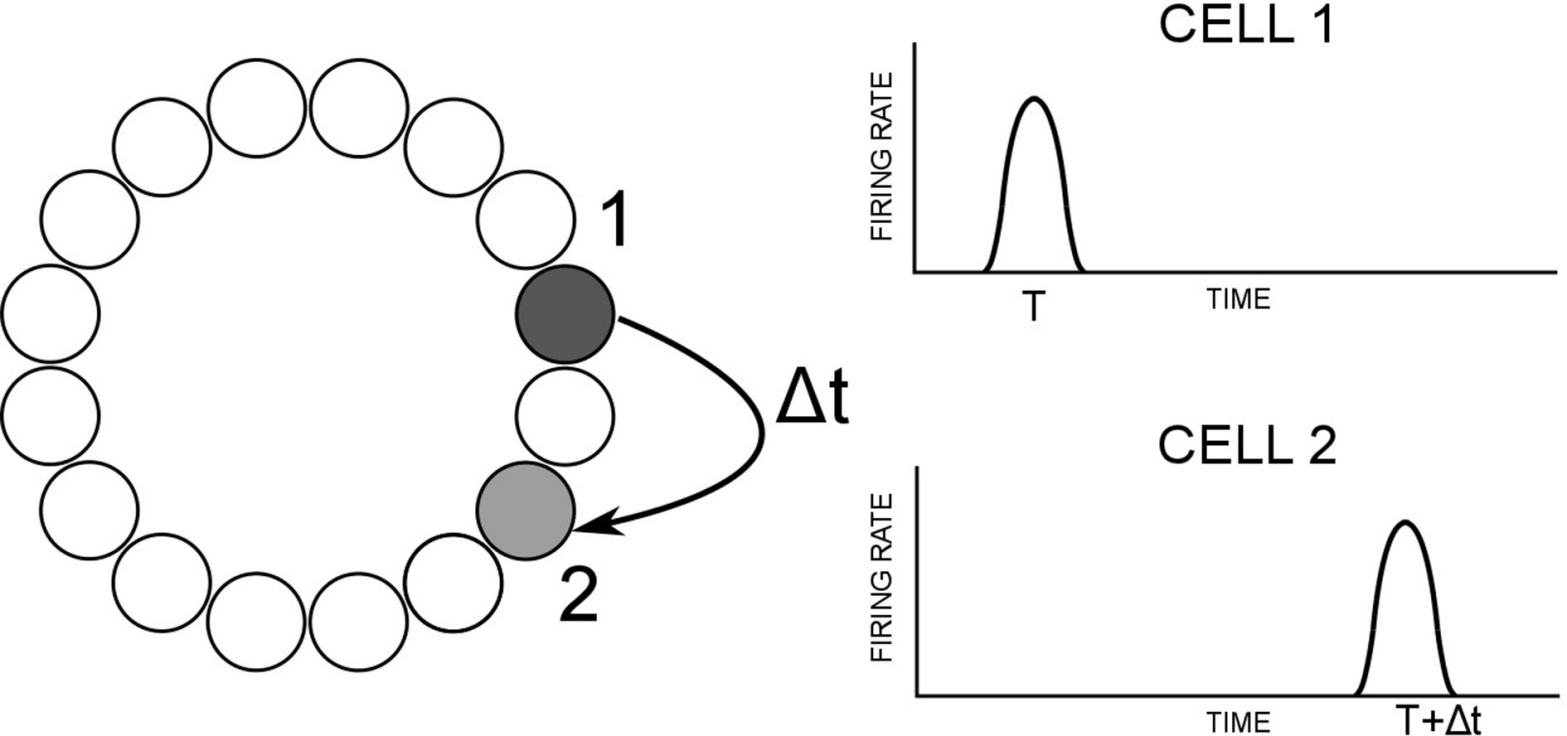
**Hypothesized path integration mechanism (left) and resulting firing times of HD cells (right)**. Cell 1 projects most strongly to cell 2, with delay Δ*t*. If cell 1 is active at time *t*, cell 2 will therefore become active at time *t* + Δ*t*. The angular separation between cells 1 and 2 will therefore govern the observed activity packet speed i.e., how far the activity packet has moved after a delay of Δ*t*.

The model used in this paper is based on the path integration mechanism of Walters et al. (2013), and is pictured in Figure 1. It consists of a ring of HD cells influencing each other via recurrent connectivity containing an axonal conduction delay Δ*t*. It can operate either as a system that self-organizes through training, or as pre-wired system with ideal connectivity. We present results from both cases.

During training, in the case of the self-organizing network, activity moves through the HD layer at constant velocity V. There is a delay Δ*t* in the learning rule, such that recurrent connections will be strengthened between post-synaptic cells and a pre-synaptic cell Δ*t* in the past. The resulting asymmetric connectivity profile will be such that a given pre-synaptic cell will project most strongly to the post-synaptic cell representing the location of the activity packet after starting at the pre-synaptic cell and moving through the HD cell layer at velocity *V* over a time interval of Δ*t*.

During testing, pre-synaptic neurons influence post-synaptic neurons via recurrent collateral connections, which contain a conduction delay Δ*t*. Given the learned profile of connectivity, pre-synaptic cells will drive up post-synaptic cells at the correct location based on the training speed and the conduction delay. A given post-synaptic cell will become active Δ*t* after its maximal projecting pre-synaptic cell, giving a test packet speed

$$\text{packet speed}=\left(\left|x_{i}-x_{j}\right|\right)/\Delta t$$

where |*x_i_* − *x_j_*| is the difference in preferred firing directions *x*_*i*_ and *x*_*j*_ of post- and pre-synaptic HD cells *i* and *j*, respectively. Given that the angular separation of cells *i* and *j* has either been learned or pre-wired on the basis of a particular training velocity, this velocity will be replicated during testing.

Path integration during testing has only reached a maximum of 81% accuracy in previous work utilizing this mechanism (Walters and Stringer, 2010; Walters et al., 2013). This paper presents a detailed investigation into factors limiting the accuracy of path integration speed. Here we show two potential sources of error: neuronal rise time and symmetric recurrent connections within neuronal layers.

### 1.2. Errors due to recurrent connectivity

One potential source of inaccuracy comes from within-layer symmetrical recurrent collateral connectivity, which has been used in past CANNs to stabilize HD cell activity in the dark. In such models, the layer of HD cells receives two peaked weight profiles: an offset profile representing idiothetic input required for path integration, and a non-offset profile originating from the same layer to stabilize HD activity in the dark. However, non-offset within-layer connectivity will reduce the effect of any offset weight profile projecting into that layer. The resultant weight profile will be a combination of these offset and non-offset components, and thus a given pre-synaptic cell will project most strongly to a different post-synaptic cell than in the case of a fully asymmetrical weight profile. This will change the value of |*x_i_* − *x_j_*| in the above equation, which can reduce packet speed. Results demonstrating this effect are given in Section 3.2.

### 1.3. Errors due to rise time

Another factor is the time neurons take to fire in response to input, known as rise time. Rise time would mean that even with purely asymmetrical connectivity, path integration will not be 100% accurate. The mechanism given above does not quite work, as post-synaptic cells will not begin firing instantaneously. There will instead be a short time lag, rise time, between when they first receive input and when they begin firing. This rise time, *t*_*R*_, will make conduction delay too long, as the new effective conduction delay will be Δ*t* + t_*R*_. Thus, by the time an activity packet is driven up, it should already be in a different place. Looking to the above equation, we can see that observed packet speed will be slower given the addition of rise time to conduction delay. Thus, error is introduced into path integration. If the system self-organizes a weight profile, neuronal rise time will not have an effect. This is because both pre- and post-synaptic HD cells would be driven up, dynamically, by the same visual input, generating the same rise time. Thus, neuronal rise time does not affect the system's ability to self-organize a correct weight profile. Results demonstrating this effect are given in Section 3.3.

Rise time will act in the context of a given conduction delay. The accuracy of observed packet speed is proportional to the ratio of conduction delay to the sum of the conduction delay and rise time. This relationship can be expressed as

$$\text{observed packet speed}=\left(\Delta t/\left(\Delta t+t_{R}\right)\right)\times \text{expected packet speed}$$

The coefficient Δ*t* + *t_R_* is maximized, and the difference between expected and observed packet speeds minimized, for either large Δ*t* or small *t*_*R*_. Therefore, path integration accuracy will be reduced with increasing *t*_*R*_ for a given value of Δ*t*, and with decreasing Δ*t* for a given value of *t*_*R*_. Results demonstrating this relationship between rise time and conduction delay are given in Section 3.3.1.

The neural network model in this paper utilizes a single layer of HD neurons, with recurrent connections that can contain both an offset component, representing idiothetic path integration input, and symmetric non-offset recurrent collaterals. The inclusion of non-offset recurrent collateral connectivity is shown to have a slowing effect on path integration speed, preventing accurate reproduction of a target velocity. The architectural prediction is made that HD areas of the rat brain involved in processing idiothetic signals for path integration will not contain within-layer recurrent collateral connectivity for this reason. Also demonstrated is the slowing effect of neuronal rise time, specifically in relation to axonal conduction delays. These findings represent a major contribution to understanding of time-accuracy in path integration and shed light on the architecture of the HD system.

## 2. Methods

Here we provide details of model governing equations and simulation protocol.

### 2.1. Model description and simulation

**Figure 2:**
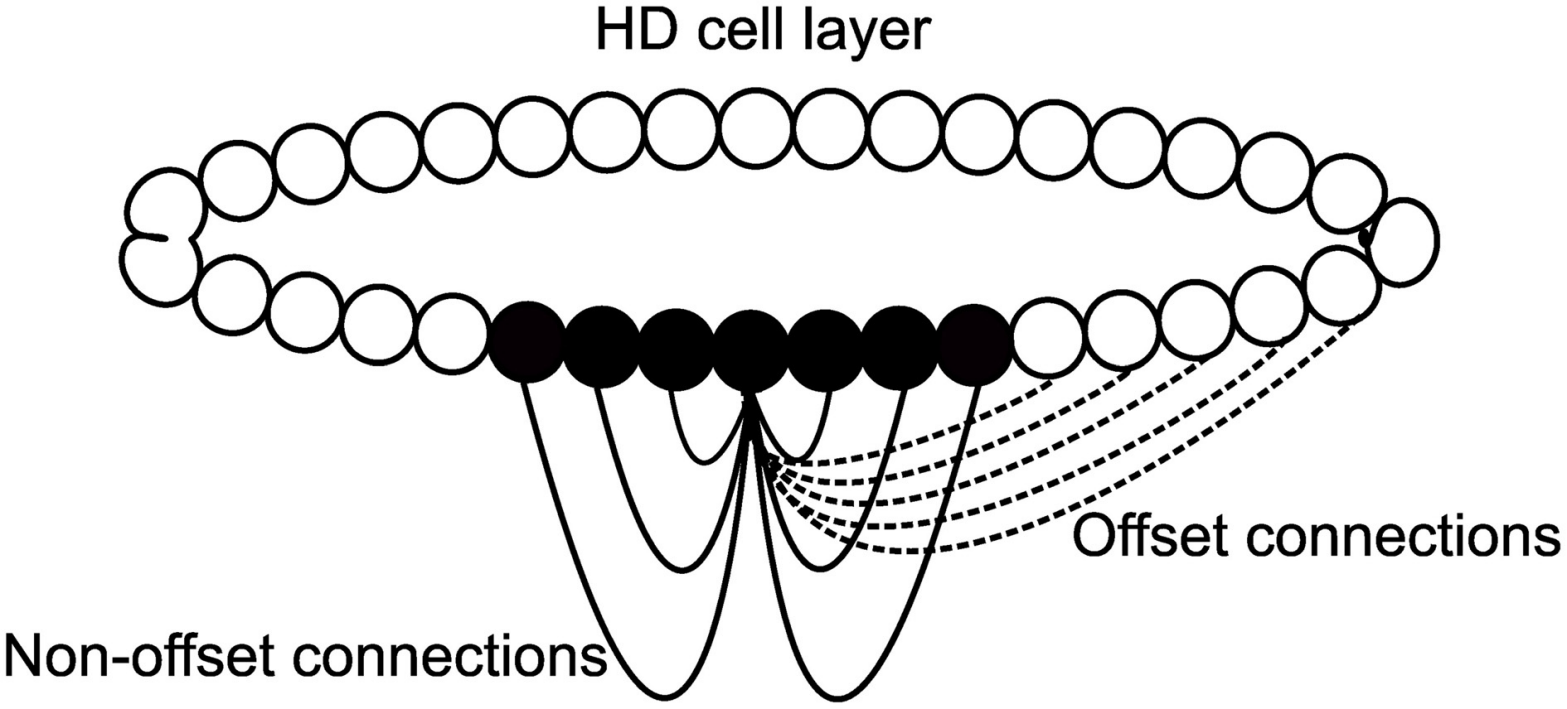
**Basic model architecture**. HD cells are connected with a recurrent weight profile that is an additive mixture of offset (dashed) and non-offset (solid) components.

The model used for this paper, based on a CANN, is pictured as a schematic in Figure 2. It consists of a single ring of HD cells. Each cell is pre-specified with a preferred firing direction, with these preferred firing directions being distributed evenly around the ring from 0 to 360°. HD cells are arranged topographically: cells with similar preferred firing directions are located next to one another. Thus, a Gaussian packet of activity in the HD cell layer represents the current HD of the agent. Individual HD cells are modeled using a leaky-integrator firing rate model, described in more detail below. Individual spikes or spike trains are not modeled, and instead neuronal firing is represented as an instantaneous average firing rate for each cell.

All cells influence one another via excitatory recurrent collateral connections. Recurrent weights are pre-wired with Gaussian profiles to have either an offset profile or a combination of an offset profile with an added non-offset component of varying strength. These are constructed in a Gaussian configuration: offset weights have an included term, such that each cell projects maximally not to itself but to an offset cell in the same layer. This offset acts to drive activity through the network at a fixed speed, intended to match a target speed, *V*°/*s*. In some simulations, this offset profile will have added to it a non-offset Gaussian weight profile. This additive profile is initialized in the same way, but is centered so that each cell will project maximally back onto itself. It should be noted then, that the combined profile from adding an offset and non-offset weight profile will result in a profile where individual cells project maximally neither onto themselves or onto the expected cell given the offset weight profile alone, but rather onto an intermediate cell between these two locations. The exact location of this intermediate cell is determined by the relative strength of the offset and non-offset weight components.

Recurrent collateral connections contain a delay Δ*t*. This represents a delay inherent in axonal conduction, the time it takes for action potentials to be conveyed along the length of an axon from near the soma to axon terminals. Incorporating an axonal conduction delay into the model means that post-synaptic cells are activated by pre-synaptic firing only after a time delay of Δ*t*. This delay is crucial for the model to shift a packet of activity around the HD ring.

### 2.2. Model equations

The activation level *h*^HD^*_i_* of HD cell *i* is given by

(1) $$\begin{array}{l} \tau^{HD}\frac{dh_{i}^{HD}(t)}{dt}=-h_{i}^{HD}(t)+e_{i}(t)\\ \;-\frac{1}{N^{HD}}\sum_{j}{\tilde{w}}^{HD}r_{j}^{HD}(t)\\ \;+\frac{\phi_{1}}{C^{HD\to HD}}\sum_{j}w_{ij}^{1}(t)r_{j}^{HD}(t-\Delta t) \end{array}$$

where *h^HD^_i_*(*t*) is the activation level of HD cell *i* at time *t*. The term −*h^HD^_i_*(*t*) represents a decay term, whereby in the absence of pre-synaptic input, the activation of HD cell *i* will fall to zero as determined by the time constant τ^*HD*^. The term *e_i_*(*t*) represents external visual input to HD cell *i* at time *t* − Δ*t*. This input term is used to generate an initial packet within the HD cell layer. It is determined by a Gaussian profile described in the simulation protocol. The term $\frac{1}{N^{HD}}\sum_{j}{\tilde{w}}^{HD}r_{j}^{HD}(t)$ represents inhibitory feedback within the HD cell layer, summed over all pre-synaptic HD cells *j*.$\tilde{w}$^*HD*^ is a global constant scaling the effect of inhibitory interneurons, and *N^HD^* is the total number of HD cells in the layer. The term $\frac{\phi_{1}}{C^{HD\to HD}}\sum_{j}w_{ij}^{1}\left(t\right)r_{j}^{HD}\left(t-\Delta t\right)$ represents excitatory input from the HD cell layer back onto itself via recurrent collateral synapses with an axonal conduction delay of Δ*t*, summed across all pre-synaptic HD cells connected to cell *i*. It consists of *r^HD^_j_*(*t*), the firing of pre-synaptic cell *j* at time *t*, and *w*^1^*_ij_*(*t*), the synaptic weight from pre-synaptic HD cell *j* to post-synaptic HD cell *i* at time *t*. It is important to note that this weight is not updated during simulation, and thus remains at the same initial value throughout. The entire term is scaled by the factor $\frac{\phi_{1}}{C^{HD\to HD}}$, which controls the overall strength of recurrent synapses with the HD cell layer where ϕ_1_ is a constant and *C*^*HD* → *HD*^ is the number of synapses each post-synaptic HD cell receives from pre-synaptic HD cells.

The firing rate *r*^HD^*_i_*(*t*) of HD cell *i* at time *t* is calculated as the hyperbolic tangent function of the activation level of cell *i* at time *t*. This function is naturally bounded in the interval [-1, 1]. However, real neurons cannot have a firing rate of <0. Thus, firing rate is bounded at zero, giving a firing rate equation of

(2) $$r_{i}^{HD}(t)=\;\{\begin{array}{ll} tanh\left(h_{i}^{HD}\left(t\right)\right) & \text{if }tanh\left(h_{i}^{HD}\left(t\right)\right)\geq 0\\ 0 & \text{otherwise} \end{array}$$

Recurrent collateral weights from HD cells back onto the HD cell layer are pre-wired with mild variations depending on whether they are to be purely offset or a mixture of offset and non-offset components. Offset weights are initialized using the Gaussian function

(3) $$w_{ij}^{1}=e^{-{\left(s_{ij}^{RC}\right)}^{2}/2{\left(\sigma^{RC}\right)}^{2}}$$

where *w*^1^_*ij*_ represents the synaptic weight from pre-synaptic HD cell *j* onto post-synaptic HD cell *i* and σ^*RC*^ is the standard deviation of the Gaussian profile. *s^RC^_ij_* is the difference between the preferred HDs *x*_*i*_ and *x*_*j*_ of the post- and pre-synaptic HD cells *i* and *j*, respectively. It is given by

(4) $$s_{ij}^{RC}=MIN\left(\left|x_{i}-\left(x_{j}+O\right)\right|,360-\left|x_{i}-\left(x_{j}+O\right)\right|\right)$$

which creates a wrap-around effect, with weights between HD cells as a population remaining continuous across the 360/0°divide. Note that the pre-synaptic preferred directions are incremented by a fixed offset, *O*. This offset *O* is calculated based on the target packet speed *V*°/*s* and the axonal conduction delay Δ*t* as

(5) O=VΔt

which effectively pre-wires connectivity with an offset that matches the amount by which a packet ought to have moved over the course of the conduction delay, given a particular target packet speed.

In some simulations, the weight profile is initialized with a combination of offset and non-offset components. In this case, the resulting weight profile is a simple addition of both offset weights, as calculated above, and non-offset weights. These non-offset recurrent collateral weights are pre-wired using the same method, with the change that pre-synaptic preferred firing directions are no longer incremented by an offset, such that the previous equation is now calculated as

(6) $$s_{ij}^{RC}=MIN\left(\left|x_{i}-x_{j}\right|,360-\left|x_{i}-x_{j}\right|\right)$$

The relative strengths of the offset and non-offset weight components are modified by the parameter λ^*NO*^, which acts as a multiplier on the non-offset weight component. Thus, the λ^*NO*^ parameter can be thought of as determining the degree of asymmetry via its effect on the non-offset weight component: as λ^*NO*^ decreases, the overall asymmetry of the weight profile tends toward the offset *O*. In both situations (offset weights and mixed weights), the weight profile is normalized after initialization. This is achieved by ensuring that the square root of the sum of the squares of afferent weights, for a particular post-synaptic cell *i*, is limited to 1. This is computed as

(7) $$\sqrt{{\sum_{j}\left(w_{ij}^{1}\left(t\right)\right)}^{2}}=1$$

In cases where an offset weight profile must be self-organized, synaptic weights are updated every timestep according to a local associative Hebbian learning rule

(8) $$\frac{dw_{ij}^{1}(t)}{dt}=kr_{i}^{HD}\left(t\right)r_{j}^{HD}\left(t-\Delta t\right)$$

where *w*^1^_*ij*_ is the synaptic weight from pre-synaptic HD cell *j* with firing rate *r^HD^_j_* onto post-synaptic HD cell *i* with firing rate *r^HD^_i_*, and *k* is the learning rate constant, which determines speed of weight change.

The differential equations given for this model cannot be solved analytically. Instead, they are implemented in the computer model by making discrete approximations of their solutions. A Forward Euler finite difference scheme is used to approximate all differential equations during simulation, and the value of the forward Euler timestep size used, δ*t* is given in Tables 1, 2.

**Table 1:** **Network parameter values for pre-wired simulations**.

**Network parameters**	
Head direction cells	500
*C*^*HD* → *HD*^	500
ϕ_1_	200.0
σ^*RC*^	10.0 °
τ^*HD*^	0.001 s
$\tilde{w}$^*HD*^	0.005
δ*t*	0.0001 s
λ^*HD*^	10.0
σ^*HD*^	20.0 °
*V*	180.0 °/*s*
Δ*t*	0.01 s

*All simulations, except where explicitly stated, are run using these parameters. All measures of time are given in seconds*.

**Table 2:** **Network parameter values for self-organizing simulations**.

**Network parameters**	
Head direction cells	500
Training time	298.5 s
Testing time	2.0 s
*C*^*HD* → *HD*^	500
ϕ_1_	60.0
τ^*HD*^	0.001 s
$\tilde{w}$^*HD*^	0.01
*I^FF^*	50.0
δ*t*	0.0001 s
λ^*HD*^	70.0
σ^*HD*^	30.0°/*s*
*V*	180.0°/*s*
Δ*t*	0.01 s
*k*	0.01

*All simulations, except where explicitly stated, are run using these parameters. All measures of time are given in seconds*.

### 2.3. Simulation protocol

At the beginning of each simulation, firing rates *r*_*i*_ of all HD cells are set to zero. If the network is self-organizing, recurrent collateral weights are initialized to a uniform negligibly small value of *w_ij_* = 0.0001. Simulation is then split into two phases: training and testing. During the training phase, a Gaussian packet of external activation is applied to the HD cell layer through the input *e_i_*(*t*). This packet of activation moves through the ring clockwise at a constant velocity of *V*°/*s*. During this phase, cell activations and firing rates are updated as in Equations (1) and (2), and recurrent collateral weights are updated and normalized as according to the Equations (7) and (8). After this training phase, the external input *e_i_*(*t*) is removed. The test phase then begins, with the network continuing to update in the absence of any external input. The recurrent collateral weight profile is fixed during the test phase, and thus no further changes in synaptic strengths occur. In some simulations, a symmetric non-offset weight profile is overlaid onto the existing learned weight profile at the beginning of the test phase. This is done identically to pre-wired simulations, as described in Equation (6).

If the network is not self-organizing, recurrent collateral connections are pre-wired as offset or a mixture of offset and non-offset, as detailed in Equations (3–6). Synaptic weights are then normalized as in Equation (7). An external input, *e*_*i*_ in Equation (1), is applied for 200 *ms*, and both the activation *h^HD^_i_*(*t*) and firing rate *r^HD^_i_*(*t*) of all HD cells are updated according to Equations (1) and (2). *e*_*i*_ is calculated for each HD cell *i* by a Gaussian profile

(9) $$e_{i}=\lambda^{HD}e^{-{\left(s_{i}^{HD}\right)}^{2}/2{\left(\sigma^{HD}\right)}^{2}}$$

where λ^*HD*^ is a scaling factor determining the strength of this input to HD cell *i* and σ^*HD*^ is the standard deviation of the Gaussian profile. *s^HD^_i_* is the difference between the true HD *x* and the preferred direction *x*_*i*_ of post-synaptic HD cell *i*. It is given by

(10) $$s_{i}^{HD}=MIN\left(\left|x_{i}-x\right|,360-\left|x_{i}-x\right|\right)$$

which creates a wrap-around effect, with response profiles of HD cells as a population remaining continuous across the 360/0°divide.

This input acts solely to initialize an activity packet at location *x* within the HD cell layer. After 200*ms*, external input is removed, and the network is simulated for a further 2*s*. During this latter phase, HD cell activations and firing rates continue to be updated as in Equations (1) and (2). It is important to note that, during this second phase of simulation, HD cell activity is sustained solely through recurrent collateral activity. The speed at which the HD cell activity packet moves in this latter phase is the critical behavior to be observed, and to be compared to the target speed *V*.

## 3. Results

### 3.1. Data analysis

#### 3.1.1. Speed

In order to calculate the speed of motion of the HD layer activity packet, the center of mass (i.e., location) of HD layer firing rates is computed at each forward Euler timestep. This was calculated using an established population vector scheme (Georgopoulos et al., 1986; Song and Wang, 2005) and is as follows

(11) $$\theta_{pop}(t)=\arctan\left(\frac{\sum_{i}r_{i}\sin\left(\theta_{i}\right)}{\sum_{i}r_{i}\cos\left(\theta_{i}\right)}\right)$$

where θ*_i_* is the preferred direction of HD cell *i* with firing rate *r*_*i*_.

However, because the ring effectively wraps around, it can provide problems for taking the mean of a set of locations spanning the 360/0° mark. For example, the mean of three cells firing maximally at 310°, 330°, and 350° would be correctly calculated by the above formula as 330°. However, the mean of three cells firing maximally at 350°, 10°, and 30° would be incorrectly calculated as 130°, rather than the correct value of 10°. To account for this, the following corrected formula is used instead

(12) $$\begin{array}{l} \;\arctan\left(\frac{\sum_{i}r_{i}\sin\;(\theta_{i})}{\sum_{i}r_{i}\cos\;(\theta_{i})}\right)\text{ if}\sum_{i}r_{i}\sin\;(\theta_{i})>0,\\ \;\sum_{i}r_{i}\cos\;(\theta_{i})>0;\\ \theta_{pop}(t)=\arctan\left(\frac{\sum_{i}r_{i}\sin\;(\theta_{i})}{\sum_{i}r_{i}\cos\;(\theta_{i})}\right)+180\\ \text{ if}\sum_{i}r_{i}\cos\;(\theta_{i})<0;\\ \;\arctan\left(\frac{\sum_{i}r_{i}\sin\;(\theta_{i})}{\sum_{i}r_{i}\cos\;(\theta_{i})}\right)+360\\ \text{ if}\sum_{i}r_{i}\sin\;(\theta_{i})<0,\sum_{i}r_{i}\cos\;(\theta_{i})>0; \end{array}$$

#### 3.1.2. Weight offset

We set the offset of the asymmetric weight component to a known value in pre-wired simulations. However, in cases where a non-offset component is added to the offset component, the new effective offset must be calculated. This is also true of self-organizing simulations, where the offset is unknown and develops as a result of training. In order to calculate the weight offset in the efferent recurrent weights for individual HD cells, a similar center of mass, referred to as the weight vector *x^w^_j_*, is computed for the recurrent weight profile at various points during simulation. This is done similarly to the activity packet population vector scheme, but using the recurrent weight profile from the perspective of a projecting HD cell *j*. Thus, the weight vector tells us the overall direction to which the projecting weights point for a given pre-synaptic cell. The center of mass of the weight vector *x*^*w*^_*j*_ is calculated for each pre-synaptic cell *j* as

(13) $$x_{j}^{w}(t)=\arctan\left(\frac{\sum_{i}w_{ij}\sin\;(x_{i})}{\sum_{i}w_{ij}\cos\;(x_{i})}\right)$$

where the sum is over pre-synaptic HD cells *i*.

Again, a corrected forumula (Batschelet, 1981; Fisher, 1993) was used to account for data circularity

(14) $$\begin{array}{l} \;\arctan\left(\frac{\sum_{i}w_{ij}\sin\;(x_{i})}{\sum_{i}w_{ij}\cos\;(x_{i})}\right)\text{ if}\sum_{i}w_{ij}\sin\;(x_{i})>0,\\ \;\sum_{i}w_{ij}\cos\;(x_{i})>0;\\ x_{j}^{w}(t)=\arctan\left(\frac{\sum_{i}w_{ij}\sin\;(x_{i})}{\sum_{i}w_{ij}\cos\;(x_{i})}\right)+180\\ \text{ if}\sum_{i}w_{ij}\cos\;(x_{i})<0;\\ \;\arctan\left(\frac{\sum_{i}w_{ij}\sin\;(x_{i})}{\sum_{i}w_{ij}\cos\;(x_{i})}\right)+360\\ \text{ if}\sum_{i}w_{ij}\sin\;(x_{i})<0,\;\sum_{i}w_{ij}\cos\;(x_{i})>0;\; \end{array}$$

where *w_ij_* is the strength of the synapse from post-synaptic HD cell *j* onto post-synaptic HD cell *i* and each post-synaptic cell has the preferred firing direction *x*_*i*_.

The new offset value is then calculated using the final projecting weight vector *x^w^_j_* and the preferred firing direction *x*_*j*_ for each pre-synaptic cell *j*

(15) $$O_{new}=MIN\left(\left|x_{j}^{w}-x_{j}\right|,360-\left|x_{j}^{w}-x_{j}\right|\right)$$

which, similarly to activity packet speed calculations, accounts for the circular nature of the data.

### 3.2. Non-offset symmetric within-layer recurrent connectivity slows path integration

**Figure 3:**
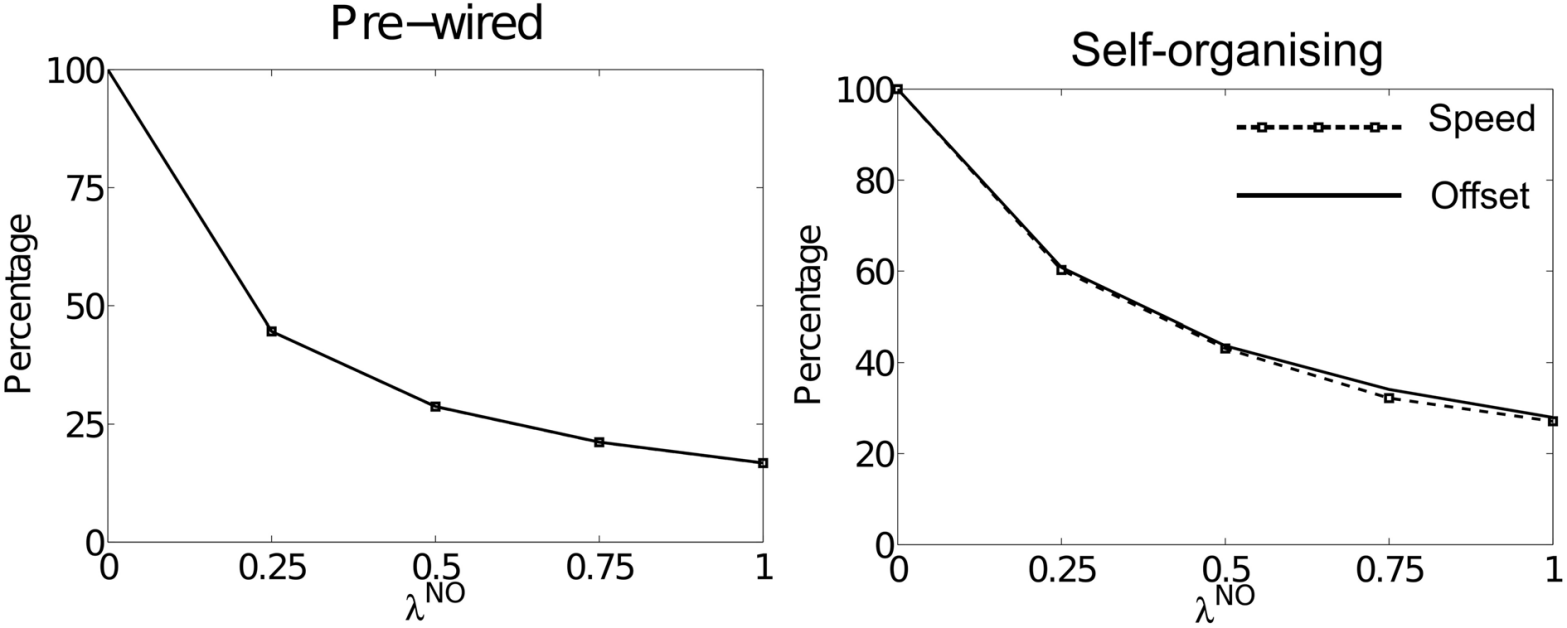
**The effects of altering λ^*NO*^ on packet speed (black dashed line with square markers) and average HD layer offset (solid black line) as a percentage of their respective values at λ^*NO*^ = 0**. For both pre-wired (left) and self-organizing networks (right) these two lines correspond, showing that introduction of within-layer non-offset connectivity always causes a proportional reduction in path integration speed.

Figure 3 shows summary results for simulations run using offset connectivity overlaid with non-offset connectivity of varying strengths (λ^*NO*^). Both packet speed (dashed line with square markers) and average HD layer offset (solid line) are plotted as a percentage of that observed at λ^*NO*^ = 0. As λ^*NO*^ increases we see a corresponding reduction in packet speed, which is directly proportional to the reduction in average offset. This is true of both pre-wired (left) and self-organizing (right) simulations. Thus, it seems that the addition of a symmetric non-offset weight profile limits packet speed during path integration.

Figure 1 shows speed and offset as a percentage of their values during simulations with λ^*NO*^ = 0 rather than as a percentage of the target velocity. However, in both cases the highest value packet speed at λ^*NO*^ = 0 does not match the target velocity of 180°. Even with perfectly pre-wired offset weights, top speed is only 165.14°: 91.8% of the target velocity. In the self-organizing simulations top speed was 162.26° (90.1%). This suggests that another factor limits packet speed.

### 3.3. Rise time slows path integration

**Figure 4:**
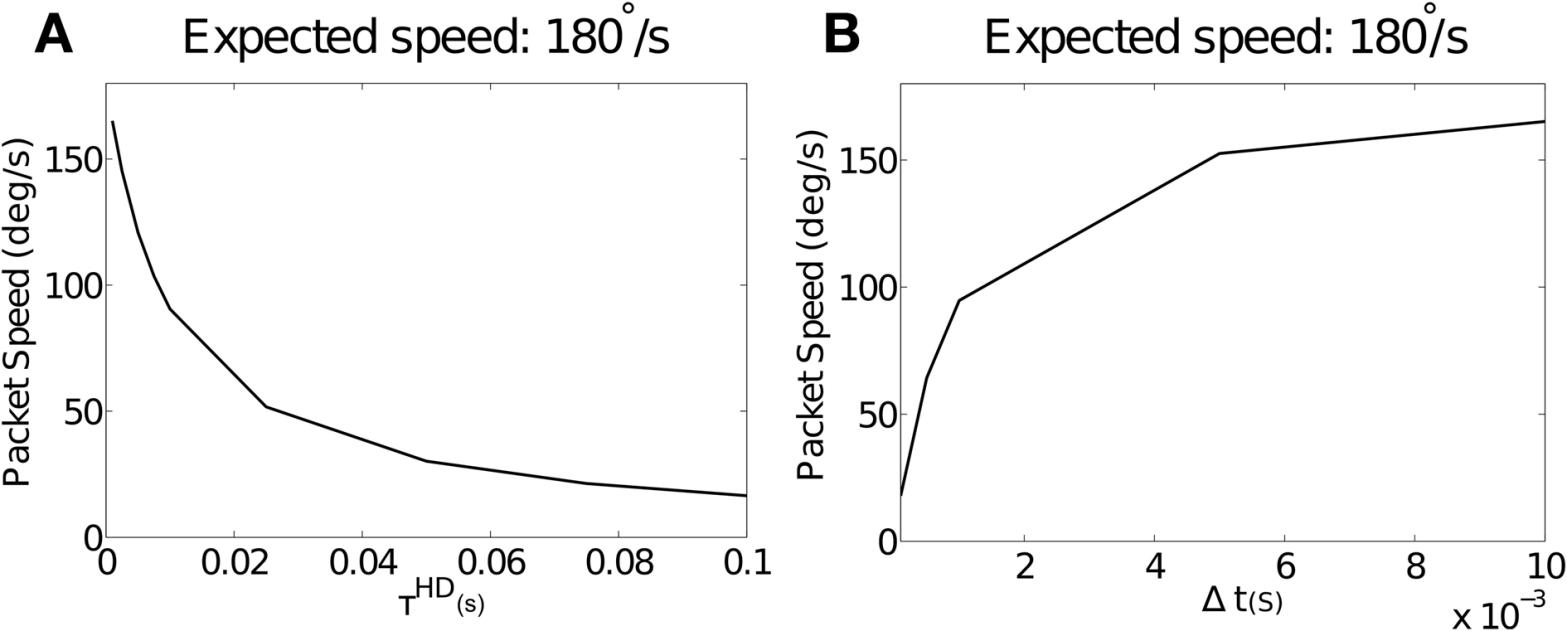
**(A)** The effect of increasing values of τ^*HD*^ on packet speed with constant Δ*t* = 0.01. As τ^*HD*^ increases, and with it neuronal rise time, there is a reduction in packet speed. As τ^*HD*^ tends to zero, the packet speed tends to the expected speed of 180°. **(B)** The effect of varying values of the axonal conduction delay Δ*t* on packet speed with a constant value of τ^*HD*^ = 0.001. As Δ*t* gets smaller relative to rise time, packet speed is reduced. As Δ*t* increases, the packet speed tends to the expected speed of 180°.

Figure 4A shows simulation results with varying values of the simulated neuronal time constant τ^*HD*^, which directly affects the rise time of individual neurons, and λ^*NO*^ = 0. Packet speed is plotted over all values of τ^*HD*^ for the pre-wired model. Conduction delay is held constant at Δ*t* = 0.01. Increasing the value of τ^*HD*^, and thus increasing rise time, reduces packet speed. This trend was near-identical in the self-organizing model. In this case, τ^*HD*^ did not affect self-organization, with offset remaining constant with changing rise time (not shown).

#### 3.3.1. Rise time acts in the context of axonal conduction delay

We hypothesize that the time taken for pre-synaptic neurons to drive up a post-synaptic neuron will be too long by the amount that post-synaptic neuronal rise time adds to the axonal conduction delay. If this is the case, a constant rise time should be more or less severe in the context of varying Δ*t*. Figure 4B shows packet speeds from simulations run with a constant value of τ^*HD*^ = 0.001 and varying values of Δ*t*.

Consistent with this hypothesis, we see that packet speed is slowest for small values of Δ*t*. This is because a constant error introduced by rise time is proportionally larger in relation to short conduction delays. Varying target speed with constant Δ*t* and τ^*HD*^(results not shown) did not affect packet speed as a proportion of the target.

## 4. Discussion

In this paper we identify, through simulation, two key hypothesized sources of error in path integration. Firstly, the antagonism between symmetrical (non-offset) recurrent collateral weights and asymmetrical (offset) weights. Secondly, neuronal rise time. Both of these factors are investigated in two variants of the model: one with pre-wired synaptic weights and one which undergoes training and accompanying self-organization of the offset weight profile.

Here we show that packet speed is reduced in direct proportion to the strength of the non- offset weight component, which represents symmetrical recurrent collateral connectivity; a key aspect of most continuous attractor neural networks (CANNs). We therefore hypothesize that individual layers of the HD system, which play a dominant role in combining allothetic and idiothetic signals to perform path integration, do not contain recurrent connections to help stabilize activity packets representing current HD, because these connections would slow down path integration as soon as the animal begins to rotate. The fundamental problem is that the recurrent connections within a layer could only learn one specific rotation speed, whether this is either no rotation or some fixed speed of rotation. If the animal rotates at any other speed, these recurrent connections will introduce error into the path integration. Specifically, error will be introduced if signals for two different speeds (e.g., current head velocity and no head velocity) co-occur in time, a necessary consequence of within-layer recurrent connectivity.

Following the attractor hypothesis of Skaggs et al. (1995), the majority of extant HD cell system models are based on CANN architectures and therefore employ such connectivity (Touretzky, 2005). CANN-based models have been used to explain a large range of HD cell phenomena, such as their ability, provided the rat continues to face in the cells preferred direction, to fire persistently without adaptation (Taube, 1998). However, the fact that symmetrical connections have been shown in this model system to impede path integration speed suggests a major shift for HD modeling approaches away from the current form of CANN architectures. Certainly, there has been a conspicuous lack of any hard evidence for recurrent collateral connectivity in HD cell regions such as the lateral mammillary nucleus (Allen and Hopkins, 1989) and anterodorsal thalamic nuclei (Rubin et al., 2001).

What, then, are the alternatives? We suggest that between-layer rather than within-layer connectivity is crucial. This is based on neurophysiological evidence demonstrating reciprocal connectivity between excitatory lateral mamilliary nucleus (LMN) cells and inhibitory dorsal tegmental nucleus (DTN) cells (Allen and Hopkins, 1988, 1989); with this LMN-DTN complex being a likely candidate substrate for the generation of the HD signal (Taube and Bassett, 2003; Song and Wang, 2005; Taube, 2007).

**Figure 5:**
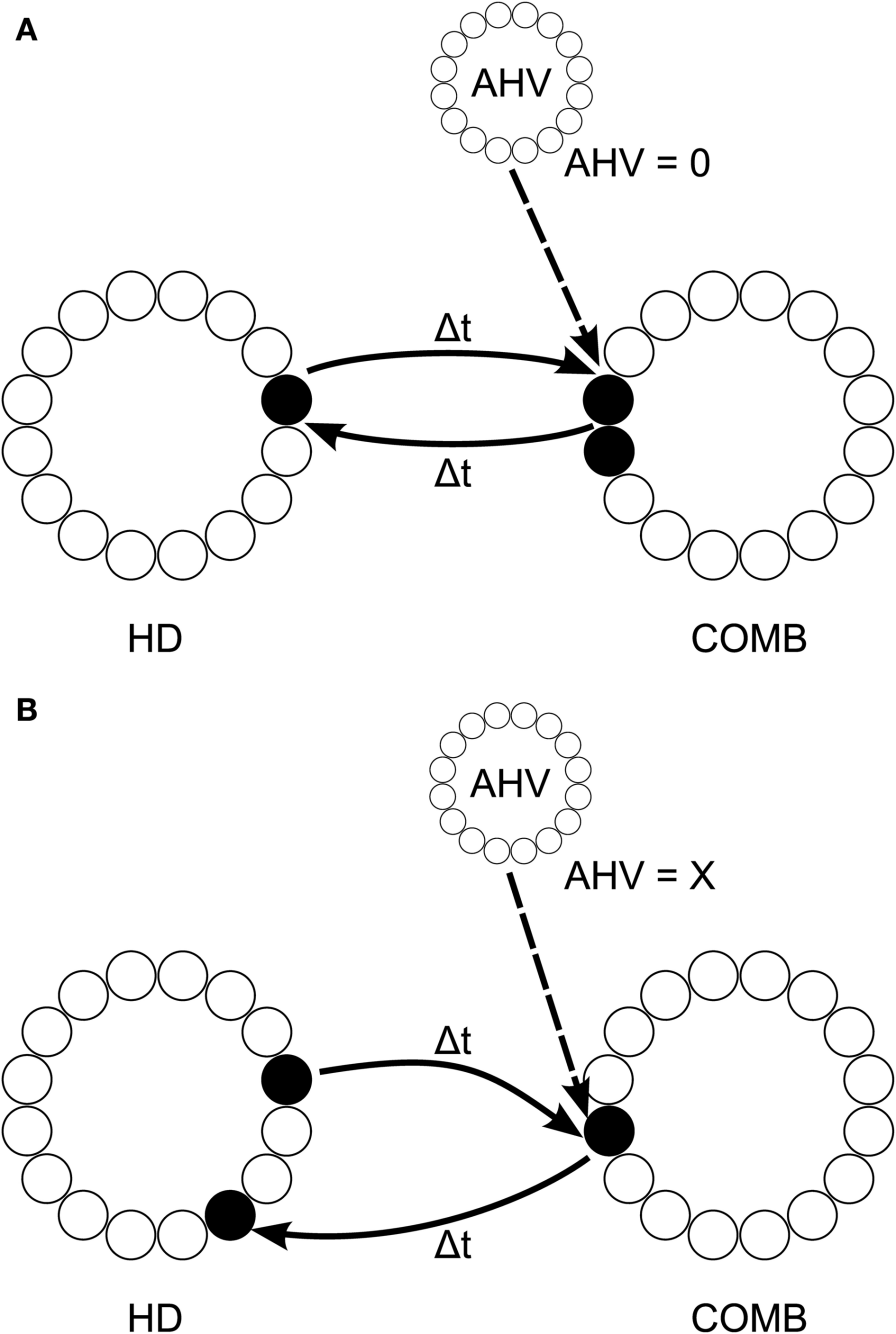
**Proposed model architecture, with HD cell activity both sustained and shifted by connectivity incoporation axonal conduction delays Δ*t***. This connectivity is reciprocal with a layer of combination (COMB) cells, which learn to respond to a particular combination of HD and AHV through competitive learning. During no head rotation **(A)**, HD activity is stabilized through reciprocal connectivity with COMB cells representing the combination of current head direction and no angular head velocity (AHV = 0). When the head is rotating **(B)**, activity is shifted through the HD layer via connectivity with different COMB cells representing the conjunction of current head direction with angular head velocity (AHV = x, where x is some non-zero value). Connectivity from these COMB cells back onto the HD layer projects with an offset from the original HD activity of 2Δ*tx*; causing HD cell activity to accurately track true head direction given current angular head velocity.

We propose an architecture, shown in Figure 5, with a layer of LMN HD cells which are reciprocally connected to a DTN layer of combination (COMB) cells, in the manner of Walters and colleagues (Walters and Stringer, 2010; Walters et al., 2013), which fire to a combination of HD and angular head velocity (AHV). However, symmetric non-offset recurrent connections would be omitted from the HD layer. The job of stabilizing HD cell activity packets would be assumed by inputs from the COMB layer. The COMB layer would receive input signaling head rotation from angular head velocity (AHV) cells: it is known that the DTN receives an AHV signal from the nucleus prepositus hypoglossi (NPH) (Khalsa et al., 1987), which itself receives projections from vestibular nuclei (McCrea and Baker, 1985), and from the supragenual nucleus (Biazoli et al., 2006), which is critical for generating a normal head directon signal in the anterior thalamus (Clark et al., 2012). Single-cell recordings confirm that DTN contains both AHV cells and cells responding to combinations of HD and AHV (Bassett and Taube, 2001; Sharp et al., 2001).

Some AHV cells show firing even when the head is not rotating (Bassett and Taube, 2001). This would allow a subset of cells in the COMB layer to learn to respond to a combination of a specific HD represented by the LMN layer and no rotation represented by AHV cells in the NPH. These COMB cells could then form reciprocal back-connections with the same subset of HD cells in the LMN layer. This connectivity would support a stable attractor state during periods of no rotation. Other subsets of COMB cells could, using axonal conduction delays, learn to respond to a combination of a current HD and head rotation at a particular velocity. These COMB cells would form back-connections onto the HD layer with an offset determined by axonal conduction delay and current AHV. During path integration, no-rotation COMB cells would become quiescent, whilst COMB cells responding to HD and rotation would begin firing and act to shift the HD cell activity packet. This architecture would allow sustained HD cell firing without affecting path integration speed during rotation. The idea of reliance on ascending vestibular information to generate a sustained HD representation is particularly important, given that damage to the vestibular system eliminates the HD cell signal (Stackman and Taube, 1997; Stackman et al., 2002; Bassett and Taube, 2005; Valerio and Taube, 2012).

Within the COMB layer, neurons learn to respond to specific combinations of HD and AHV. At any moment, only the correct subset of COMB cells are activated corresponding to the current rotation speed. This means that the bi-directional connections between the two layers learn to encode specific rotation speeds, with different connections encoding different speeds and thus reducing error from interference of other speeds. Consequently, the connectivity between the two layers can implement accurate path integration across all of the trained speeds, including stabilizing the HD activity packet during no rotation.

We also show that a long neuronal rise time introduces error in path integration: the longer the rise time for a given conduction delay, the greater the error in path integration. Rise time also appears to reduce packet speed in relation to the axonal conduction delay used: the shorter the conduction delay for a given rise time, the more severe the slowing effect. Rise time does not appear to have an effect on the self-organization of offset connectivity. This is because both pre and post-synaptic cells are driven up dynamically by the same external input, and thus both have the same rise time. Crucially, the post-synaptic cell is driven up during training by external input rather than by the firing of the pre-synaptic cell conveyed via recurrent connections as in testing. During testing, post-synaptic cells will be driven up by the firing of pre-synaptic cells via connectivity containing a conduction delay. This conduction delay used will have added to it the post-synaptic rise time, constituting an error in path integration. Our findings support the intuition that rise time causes signal transmission between HD cells to be too long during testing but not during training, with the correct offset self-organizing even with long rise times.

Whilst rise time is a particular issue for a rate-coded neural network as in this paper, it may be ameliorated by shifting to a spiking network addressing the fine dynamics of neuronal firing. Such networks can update their representations very rapidly (Brunel and Wang, 2001). Neurons may have a background level of activation that is very close to firing threshold, thus reducing the time taken for input to drive them up to firing (Fourcaud-Trocme et al., 2003). This has been demonstrated in neural network models (Tsodkys and Sejnowski, 1995; Brunel and Wang, 2001). It could thus be the case that a spiking network will allow rise time to be minimized to an extent that no longer interferes with path integration.

This paper focuses on an architectural approach to the issue of path integration speed. Previous work involving within-layer symmetric connectivity has suggested that path integration accuracy can be improved with neuronal mechanisms. One example is short-term depression (STD) (Fung et al., 2012b). It has been shown that STD is a workable mechanism for generating real-time tracking states which could compensate for rise time effects (Fung et al., 2012a). Interestingly, such a mechanisms could account for the finding that some HD cell firing anticipates actual HD (Blair and Sharp, 1995). However, this STD effect has been shown in the “classic” CANN architecture, which is unlikely to exist in the HD cell system. We suggest a move away from such connectivity, but it remains to be shown whether this STD effect operates within a multi-layer HD network of the sort we hypothesize.

This model represents a simple, yet powerful, approach to uncovering key factors affecting accuracy of path integration speed. Several simplifications have been made in striving for maximum explanatory power. In this context, it is important to be clear about what issues we are and are not addressing. Firstly, HD cells are explicitly pre-designated with a preferred direction. In reality, the preferred direction of individual HD cells must be calibrated in some way to establish the specific connectivity, both between HD cells, and from visual areas onto HD areas. This paper however, is not focused on answering how HD cells acquire a preferred direction but rather how mature HD cells behave during path integration.

We employ a single layer of recurrent connections with varying degrees of asymmetry. The final weight profile is an additive combination of two components. This represents two sources of input to HD cells: non-offset synapses from the HD layer back onto itself, and offset output of some path integration system. Reducing these two sources to one layer demonstrates clearly their interaction. It is believed that the case in which HD cells receive inputs from different sources would not be qualitatively different provided they occur at the same time.

All simulations reported here were run with a target velocity of *V* = 180.0°/*s*. This was considered fair given previous anaylsis of rat AHV as lying in the range of 0–1000°/*s* (Stackman and Taube, 1998; Bassett and Taube, 2001; Sharp et al., 2001). There is however, no reason why the effects presented in this paper will not be seen at different velocities. Indeed, simulations were carried out with a variety of values of *V* in the range 45–360°/*s*. Although this is smaller than the experimentally-observed range of rat angular head velocities, we note that our simulations use a continuous rotation at a constant speed over a period of time, a movement which a rat is unlikely to perform at the limits of possible rotation velocities. An interesting further investigation might well be to extend these simulations to more complex head rotations at multiple speeds in multiple directions.

Here we investigate the issue of path integration speed in continuous attractor models of the HD cell system. Two major factors were discovered to affect the replay speed of path integration systems: the presence of within-layer symmetric non-offset recurrent collateral connectivity, and neuronal rise time. In the case of rise time, it appears that this factor does not adversely affect the self-organization of these path integration systems. We show that it is possible to have perfect path integration accuracy if rise time is negated and within-layer connectivity is purely offset. These findings represent a major contribution to theoretical understanding of the factors governing accuracy of path integration and result in key architectural predictions. Future approaches to coping with these speed-limiting factors are suggested.

## Author contributions

Hector J. I. Page, Simon M. Stringer, and Daniel Walters designed the model and simulation protocol, discussed results, and commented on the manuscript. Hector J. I. Page and Simon M. Stringer wrote the manuscript, Hector J. I. Page and Daniel Walters revised the manuscript, Hector J. I. Page programmed simulations and analyzed results.

### Conflict of interest statement

The authors declare that the research was conducted in the absence of any commercial or financial relationships that could be construed as a potential conflict of interest.
